# Draft genome sequence of three *Glaciozyma watsonii* strains isolated from near the Syowa station area, East Antarctica

**DOI:** 10.1128/mra.00831-25

**Published:** 2025-09-12

**Authors:** Masaharu Tsuji, Jun-ichi Ishihara, Hiroki Takahashi, Yasuhiro Gotoh, Tetsuya Hayashi, Sakae Kudoh

**Affiliations:** 1 Department of Science of Technology Innovation, Nagaoka University of Technology 52756, Nagaoka, Niigata, Japan; 2 National Agriculture and Food Research Organization 13516, Tsukuba, Ibaraki, Japan; 3 Medical Mycology Research Center, Chiba University 12737, Chiba, Chiba, Japan; 4 Advanced Genomics Center, National Institute of Genetics 26359, Mishima, Shizuoka, Japan; 5 Department of Bacteriology, Faculty of Medical Sciences, Kyushu University, Higashi-ku 12923, Fukuoka, Japan; 6 National Institute of Polar Research 13554, Tachikawa, Tokyo, Japan; 7 Department of Polar Science, The Graduate University for Advanced Studies 13177, Tachikawa, Tokyo, Japan; Rochester Institute of Technology, Rochester, New York, USA

**Keywords:** Antarctica, yeast, draft genome sequence

## Abstract

Three strains of *Glaciozyma watsonii* were isolated from the Inhovde area, East Antarctica. This yeast is known as having ice-binding proteins and cold-active enzymes that are active, even at subzero temperatures. We report a draft genome sequence of these three *Glaciozyma watsonii* strains isolated from East Antarctica.

## ANNOUNCEMENT

The genus *Glaciozyma* was originally isolated from the Italian Alps and Antarctica (1). This yeast is known to have ice-binding proteins and cold-active serine proteases (1). Furthermore, this yeast can grow, even at −3°C, and has a remarkable ability to grow, even when inoculated into agar medium frozen at −80°C and cultured at −3°C (2). Based on these findings, the genus *Glaciozyma* is considered one of the fungi most adapted to cold temperatures. During the 56th JARE expedition, ice and snow samples were collected from the Inhovde area (69°51′S, 37°06′W). We isolated three strains of *G. watsonii*, namely, B-4-2 = NIPR-00560, 057-4-1 = NIPR-00545, and 058-4-1 = NIPR-00551 from ice samples in the Inhovde area, East Antarctica (3). Here, we report on the draft genome sequence of these three strains of *G. watsonii* isolated from Inhovde near the Syowa Station.

The yeast culture and DNA extraction methods were followed by those previously reported (4–6). Briefly, yeast cells were cultured in a yeast extract-peptone-dextrose (YPD) liquid medium (Difco) at 10°C for 10 days. The cells from cultures were collected by centrifugation at 5,000 *g* for 10 min at 4°C. The cell pellets were washed with sterile distilled water and collected by centrifugation again. The cell pellets were dried by a vacuum freeze dryer. The lyophilized cells were then powdered in a mortar and used for DNA extraction. The genomic DNA was extracted and purified using a NucleoBond AXG100 column with a Nucleo Buffer Set III (TaKaRa Bio) according to the manufacturer’s protocol. Concentration and purification of the genomic DNA were determined by NanoDrop (Thermo Scientific) and the Qubit dsDNA BR assay kits (Thermo Scientific). Then, the genomic DNA was fragmented using the microTUBE (Covaris), and the genomic libraries were constructed using the Illumina TruSeq DNA PCR-free library preparation kit (Illumina). The qualities of the libraries were confirmed using the Quant-iT PicoGreen dsDNA Assay Kit, and the library sizes were checked using the Agilent 2200 TapeStation System (Agilent Technologies, Inc.). The libraries were sequenced on the HiSeq 2500 platform (Illumina) to generate paired-end sequence reads (151 bp ×2). In this study, default parameters were used, except where otherwise noted. Adapters and low-quality bases were trimmed using fastp ver. 0.12.4 (7). Assembly of the nuclear genomes was conducted using Platanus ver. 1.2.4 (8). After the identification of repeat sequences using RepeatModeler ver. 2.0.1 and RepeatMasker ver. 4.1.2 (9), a summary of the genomic analysis results of the three *G. watsonii* strains is shown in Table 1. Briefly, the genome size ranged from 18.8 to 18.9 Mb, and the GC contents ranged from 60.5 to 60.6%.

**TABLE 1:** Assembly summary for the draft genomic data of the three *Glaciozyma watsonii* strains

	Data for each strain		
Characteristic	B-4-2	057-4-1	058-4-1
Sampling site	Inhovde	Inhovde	Inhovde
No. of raw reads	4,912,437,398	6,013,131,664	6,506,878,108
No. of scaffolds	268	293	251
Total length (Mb)	18.8	18.9	18.8
GC content (%)	60.6	60.5	60.6
Scaffold *N_50_* value (kb)	285	268	306
Coverage (×)	235.6	293.4	316.4
GenBank accession no.	BAAHZA010000000.1	BAAHZB010000000.1	BAAHZC010000000.1
DRA accession no.	DRR118805, DRR118806	DRR118807, DRR118808	DRR118809, DRR118810

*G. watsonii* has the remarkable ability to grow, even when inoculated on frozen agar medium and cultured at −3°C. Genomic information on *G. watsonii* will play an important role in elucidating the remarkable ability of this yeast to grow, even in frozen environments, but will also contribute to the search for new pharmaceutical raw materials.

## Data Availability

This Whole-Genome Shotgun project has been deposited at DDBJ/EMBL/GenBank under accession numbers BAAHZA010000000.1 (B-4-2 = NIPR-00560), BAAHZB010000000.1 (057-4-1 = NIPR-00545), and BAAHZC010000000.1 (058-4-1 = NIPR-00551). The raw reads are available under DRA accession numbers DRR118805, DRR118806 (B-4-2 = NIPR-00560), DRR118807, DRR118808 (057-4-1 = NIPR-00545), DRR118809, and DRR118810 (058-4-1 = NIPR-00551).
